# Clinical features and outcome of patients with acute respiratory failure revealing anti-synthetase or anti-MDA-5 dermato-pulmonary syndrome: a French multicenter retrospective study

**DOI:** 10.1186/s13613-018-0433-3

**Published:** 2018-09-11

**Authors:** Constance Vuillard, Marc Pineton de Chambrun, Nicolas de Prost, Claude Guérin, Matthieu Schmidt, Auguste Dargent, Jean-Pierre Quenot, Sébastien Préau, Geoffrey Ledoux, Mathilde Neuville, Guillaume Voiriot, Muriel Fartoukh, Rémi Coudroy, Guillaume Dumas, Eric Maury, Nicolas Terzi, Yacine Tandjaoui-Lambiotte, Francis Schneider, Maximilien Grall, Emmanuel Guérot, Romaric Larcher, Sylvie Ricome, Raphaël Le Mao, Gwenhaël Colin, Christophe Guitton, Lara Zafrani, Elise Morawiec, Marie Dubert, Olivier Pajot, Hervé Mentec, Gaëtan Plantefève, Damien Contou

**Affiliations:** 1 Service de Réanimation Polyvalente, Centre Hospitalier Victor Dupouy, 69 rue du Lieutenant Colonel Prudhon, 95100 Argenteuil, France; 2 Service de Réanimation Médicale, Centre Hospitalier Universitaire Pitié-Salpétrière – Assistance Publique Hôpitaux de Paris, 47-83 boulevard de l’Hôpital, 75013 Paris, France; 3 Service de Réanimation Médicale, Centre Hospitalier Universitaire Henri Mondor – Assistance Publique Hôpitaux de Paris, 51 avenue du Maréchal de Lattre de Tassigny, 94010 Créteil, France; 4 Service de Réanimation Médicale, Hôpital de la Croix-Rousse, 103 Grande rue de la Croix-Rousse, 69004 Lyon, France; 5 INSERM 955, Créteil, France; 6 Service de Médecine Intensive Réanimation, Centre Hospitalier Universitaire François Mitterrand de Dijon, 14 rue Paul Gaffarel, 21000 Dijon, France; 7 Service de Réanimation, Centre Hospitalier Régional Universitaire de Lille, 2 avenue Oscar Lambret, 59000 Lille, France; 8 Service de Réanimation Médicale, Centre Hospitalier Universitaire Bichat Claude-Bernard – Assistance Publique Hôpitaux de Paris, 46 rue Henri Huchard, 75877 Paris, France; 9 Service de Réanimation médico-chirurgicale, Centre Hospitalier Universitaire Tenon – Assistance Publique Hôpitaux de Paris, 5 rue de la Chine, 75020 Paris, France; 10 Service de Réanimation médicale, Centre hospitalier universitaire de Poitiers, 2 rue de la Milétrie, 86021 Poitiers, France; 11 Service de Réanimation médicale, Centre Hospitalier Universitaire Saint-Antoine – Assistance Publique Hôpitaux de Paris, 184 rue du Faubourg Saint-Antoine, 75012 Paris, France; 12 Service de Réanimation, Centre Hospitalier Universitaire de Grenoble Alpes, avenue Maquis du Grésivaudan, 38700 La Tronche, France; 13 Service de Réanimation médico-chirurgicale, Centre Hospitalier Universitaire Avicennes – Assistance Publique Hôpitaux de Paris, 125 rue de Stalingrad, 93000 Bobigny, France; 14 Service de Réanimation, Centre Hospitalier Universitaire de Strasbourg, 1 avenue Molière, 67200 Strasbourg, France; 15 Service de Réanimation Médicale, Centre Hospitalier Universitaire de Rouen, 1 rue de Germont, 76000 Rouen, France; 16 Service de Réanimation Médicale, Centre Hospitalier Universitaire Hôpital Européen Georges-Pompidou – Assistance Publique Hôpitaux de Paris, 20 rue Leblanc, 75015 Paris, France; 17 Service de Réanimation Médicale, Centre Hospitalier Universitaire de Montpellier, 191 avenue du Doyen Gaston Giraud, 34000 Montpellier, France; 18 Service de Réanimation Polyvalente, Centre Hospitalier Robert-Ballanger, Boulevard Robert Ballanger, 93600 Aulnay-sous-Bois, France; 19 Service de Réanimation médicale, Centre Hospitalier Régional Universistaire de Brest, Site La Cavale Blanche, Boulevard Tanguy Prigent, 29200 Brest, France; 20 Service de réanimation médico-chirurgicale, Centre Hospitalier Départemental de Vendée, Les Oudairies, 85925 La Roche sur Yon Cedex 9, France; 21 Service de Réanimation médico-chirurgicale, Centre Hospitalier du Mans, 194 avenue Rubillard, 72037 Le Mans, France; 22 Service de Réanimation médicale, Hôpital Saint-Louis, Assistance Publique Hôpitaux de Paris, 1 avenue Claude Vellefaux, 75010 Paris, France; 23 Unité de Réanimation et de Surveillance continue, Service de Pneumologie et Réanimation médicale, Groupe hospitalier Pitié-Salpêtrière, 47-83 bd de l’hôpital, 75651 Paris, France; 24 Service d’Immunologie Clinique, Hôpital Saint-Louis, Assistance Publique Hôpitaux de Paris, 1 avenue Claude Vellefaux, 75010 Paris, France

**Keywords:** Inflammatory myositis, Interstitial lung disease, ARDS, Acute respiratory failure, Diagnosis

## Abstract

**Background:**

Anti-synthetase (AS) and dermato-pulmonary associated with anti-MDA-5 antibodies (aMDA-5) syndromes are near one of the other autoimmune inflammatory myopathies potentially responsible for severe acute interstitial lung disease. We undertook a 13-year retrospective multicenter study in 35 French ICUs in order to describe the clinical presentation and the outcome of patients admitted to the ICU for acute respiratory failure (ARF) revealing AS or aMDA-5 syndromes.

**Results:**

From 2005 to 2017, 47 patients (23 males; median age 60 [1st–3rd quartiles 52–69] years, no comorbidity 85%) were admitted to the ICU for ARF revealing AS (*n* = 28, 60%) or aMDA-5 (*n* = 19, 40%) syndromes. Muscular, articular and cutaneous manifestations occurred in 11 patients (23%), 14 (30%) and 20 (43%) patients, respectively. Seventeen of them (36%) had no extra-pulmonary manifestations. C-reactive protein was increased (139 [40–208] mg/L), whereas procalcitonine was not (0.30 [0.12–0.56] ng/mL). Proportion of patients with creatine kinase ≥ 2*N* was 20% (*n* = 9/47). Forty-two patients (89%) had ARDS, which was severe in 86%, with a rate of 17% (*n* = 8/47) of extra-corporeal membrane oxygenation requirement. Proportion of patients who received corticosteroids, cyclophosphamide, rituximab, intravenous immunoglobulins and plasma exchange were 100%, 72%, 15%, 21% and 17%, respectively. ICU and hospital mortality rates were 45% (*n* = 21/47) and 51% (*n* = 24/47), respectively. Patients with aMDA-5 dermato-pulmonary syndrome had a higher hospital mortality than those with AS syndrome (*n* = 16/19, 84% vs. *n* = 8/28, 29%; *p* = 0.001).

**Conclusions:**

Intensivists should consider inflammatory myopathies as a cause of ARF of unknown origin. Extra-pulmonary manifestations are commonly lacking. Mortality is high, especially in aMDA-5 dermato-pulmonary syndrome.

## Background

Identifying the cause of acute respiratory distress syndrome (ARDS) is a crucial step for initiating a targeted treatment and improving prognosis [1, 2]. However, two recent studies [3, 4] showed that 8% of patients with ARDS according to the Berlin criteria [5] lacked exposure to “common” risk factors (e.g., pneumonia, acute pancreatitis, aspiration of gastric content or extra-pulmonary sepsis) with no etiology eventually retrieved in 80% of them [4]. For such atypical ARDS, a comprehensive diagnostic work-up, including specific immunologic tests, is recommended [6] so that to identify immune causes, typically amenable to specific therapeutic interventions (e.g., corticosteroids). Yet, an ancillary analysis [4] of an international, multicenter, prospective cohort study [7] reported that such immunological examinations were performed in only 5% of ARDS without common risk factors.

Anti-synthetase (AS) and anti-melanoma differentiation-associated gene 5 (aMDA-5) syndromes are near one of the other autoimmune inflammatory myopathies [8] potentially responsible for rapidly progressive interstitial lung disease leading to acute respiratory failure and ARDS [9–12]. AS and aMDA-5 dermato-pulmonary syndromes may be clinically indistinguishable one from another, with almost three-quarter of patients with aMDA-5 dermato-pulmonary syndrome exhibiting the clinical attributes of the AS syndrome [8]. When ARF is the initial presentation of AS or aMDA-5 syndromes [9–11, 13–17] or when extra-respiratory manifestations, such as muscular, cutaneous or articular signs are lacking [9, 18–22], the diagnosis is challenging, especially in the intensive care unit (ICU) setting, where many other reasons of acute respiratory failure (ARF) can be discussed. To the best of knowledge, a number of case reports of ARF revealing autoimmune inflammatory myopathies have been previously reported, but an extended case series has not been published as yet.

Therefore, we undertook this retrospective study in order to: (1) describe the clinical features and the outcome of patients admitted to the ICU for ARF revealing either an AS or an aMDA-5 dermato-pulmonary syndrome, and; (2) identify predictive factors of hospital mortality.

## Patients and methods

### Patients

We conducted a 13-year multicenter retrospective non-interventional study in 35 ICUs in France from January 1, 2005, to December 31, 2017. All patients older than 18 years were included if they met the following criteria: (1) admitted to the ICU for ARF not related to cardiogenic pulmonary edema; (2) no common ARDS risk factor, among pneumonia, acute pancreatitis, aspiration of gastric content, extra-pulmonary sepsis, multiple transfusions, major trauma, pulmonary vasculitis, drowning, severe burns, identified according to the Berlin definition [5]; (3) immunologic test performed during ICU stay, which was positive for anti-synthetase (Jo-1, PL7, PL12, OJ, EJ, KS, Zo, YRS/Tyr/Ha) or anti-MDA-5 autoantibodies; and (4) no alternative diagnosis for ARF. It is worth notifying that in the present study the diagnosis of AS or aMDA-5 dermato-pulmonary syndromes had to be made during the ICU stay. Therefore, those who had a diagnosis of AS or aMDA-5 made before ICU admission were not included.

The investigator of each participating center was responsible for the identification of the patients, either from the hospital medical reports, using the function “research the files in which the key words *MDA*-*5* or *anti*-*synthetase* or *myositis* occurs” of Microsoft Windows^®^, or through a search using the International *Classification of Diseases* (10th Revision) following codes: M608 (autoimmune myositis), M609 (myositis), M332 (polymyositis) and M331 (dermatomyositis). The clinical charts of all identified patients were anonymized before sending to the main investigators (DC and CV). Clinical charts were reviewed in order to check the inclusion criteria.

### Data collection

The following data were collected on a standardized anonymized case record form: demographic characteristics (age, gender), severity scores upon ICU admission (Sequential Organ Failure Assessment [23] and Simplified Acute Physiology Score II [24]), main comorbidities, delay between first respiratory sign and ICU admission, clinical examination (respiratory and extra-respiratory manifestations) and laboratory findings at the time of ICU admission (blood leukocytes and platelets counts, serum procalcitonine, C-reactive protein, creatine kinase and creatinine levels, PaO_2_/FiO_2_ with FiO_2_ calculated according to the following formula [25, 26]: FiO_2_ = oxygen flow in liter per minute × 0.04 + 0.21 when standard oxygen was used), radiological findings on chest X-ray and CT scan, cytological and bacteriological analyses of broncho-alveolar lavage (BAL) fluid, type of positive autoantibodies (Jo-1, PL7, PL12, OJ, EJ, KS, Zo, YRS/Tyr/Ha or aMDA-5), immunosuppressive treatments received (corticosteroids, cyclophosphamide, rituximab, basiliximab, tacrolimus, cyclosporine, methotrexate, intravenous immunoglobulins or plasma exchange), organ supports in the ICU (invasive mechanical ventilation, extra-corporeal membrane oxygenation (ECMO), renal replacement therapy, vasopressors), ICU and hospital length of stay, ICU and hospital mortality.

Written reports of chest CT scan performed at the time of ICU admission were sent to the main investigators (DC and CV) in order to individualize elementary lesions (ground-glass attenuation, alveolar consolidation, septal thickening, pleural effusion, pneumothorax, pneumomediastinum and mediastinal lymphadenopathy) and their location (lower or upper lobe predominance). Signs of lung fibrosis (honeycombing, traction bronchiectasis and reticulations) were also collected. Cytological analyses of BAL fluid collected at the time of ICU admission were reported, as well as results of open lung, skin or muscle biopsies, if performed.

### Statistical analysis

Continuous variables are reported as median [1st–3rd quartiles] and compared by the Mann–Whitney *U* test. Categorical variables are reported as counts and percentage points in groups and compared by using the Fisher’s exact test. Survival curves of patients with aMDA-5 and AS syndromes were drawn using the Kaplan–Meier method and compared using the log-rank test. All tests were two-sided, with *p* < 0.05 indicating statistical significance. The statistical analysis was performed by using the RStudio software version 0.99.441 (www.rStudio.com).

## Results

### Study population and clinical manifestations

From January 1, 2005, to December 31, 2017, 47 patients fulfilled the inclusion criteria, including 28 (60%) with AS syndrome (Jo-1 *n* = 13/28 (47%); PL7 *n* = 9/28 (32%); PL12 *n* = 4/28 (14%); EJ *n* = 2/28 (7%)) and 19 (40%) with aMDA-5 dermato-pulmonary syndrome. All the patients with aMDA-5 dermato-pulmonary syndrome were admitted after January 1, 2010. Demographical characteristics, main comorbidities and clinical manifestations are given in Table 1. Most of the patients had no comorbidity (*n* = 40/47, 85%). Median SAPSII and SOFA scores at the time of ICU admission were 35 [27–53] and 5 [3–8], respectively. The median delay between first respiratory sign and ICU admission was 21 [10–41] days. Most of the patients had central temperature > 38 °C (*n* = 27/47, 57%). Myalgia, arthralgia/arthritis and cutaneous manifestations occurred in 23% (*n* = 11/47), 30% (*n* = 14/47) and 43% (*n* = 20/47) of patients, respectively. About one-third of patients (*n* = 17/47, 36%) had no extra-pulmonary manifestation, in a similar proportion in aMDA-5 and AS groups.

**Table 1:** Demographical and clinical manifestations of patients with acute respiratory failure revealing anti-synthetase syndrome or dermato-pulmonary syndrome associated with anti-MDA-5 antibodies

	Missing data	All patients *N* = 47	aMDA-5 ARF *N* = 19	AS ARF *N* = 28	*p*
Age, years median [IQR]	0	60 [52–69]	60 [51–67]	63 [54–73]	0.51
Male, *n* (%)	0	23 (49)	8 (42)	15 (54)	0.64
SOFA score median [IQR]	0	5 [3–8]	4 [2–8]	5 [3–8]	0.77
SAPSII median [IQR]	0	35 [27–53]	34 [27–53]	38 [27–50]	0.94
Comorbidities, *n* (%)					
Chronic respiratory failure	0	1 (2)	0 (0)	1 (4)	1.00
Congestive heart failure	0	0 (0)	0 (0)	0 (0)	1.00
Cirrhosis	0	1 (2)	0 (0)	1 (4)	1.00
Chronic kidney failure	0	0 (0)	0 (0)	0 (0)	1.00
Active solid cancer or malignant hemopathy	0	0 (0)	0 (0)	0 (0)	1.00
HIV	0	0 (0)	0 (0)	0 (0)	1.00
Diabetes mellitus	0	5 (11)	1 (5)	4 (14)	0.64
Obesity (body mass index ≥ 30 kg/m^2^)	0	1 (2)	0 (0)	1 (4)	1.00
No comorbidities	0	40 (85)	18 (95)	22 (79)	0.60
Active or former tobacco use, *n* (%)	0	20 (43)	7 (37)	13 (46)	0.73
Respiratory manifestations, *n* (%)					
Delay first respiratory sign—ICU admission, days	2	21 [10–41]	21 [11–43]	21 [10–41]	0.73
Dry cough	0	23 (49)	8 (42)	15 (54)	0.64
Chest pain	0	0 (0)	0 (0)	0 (0)	1.00
Hemoptysis	0	1 (2)	0 (0)	1 (4)	1.00
Bilateral crackles	5	34 (81)	13 (77)	21 (84)	0.69
Fever (> 38 °C)	0	27 (57)	9 (47)	18 (64)	0.40
Extra-respiratory manifestations, *n* (%)					
Myalgia	0	11 (23)	2 (11)	9 (32)	0.16
Muscles weakness	0	6 (13)	1 (5)	5 (18)	0.38
Arthralgia/arthritis	0	14 (30)	6 (32)	8 (29)	1.00
Cutaneous manifestations	0	20 (43)	13 (68)	7 (25)	0.008
Mechanic’s hand	0	8 (17)	3 (15)	5 (19)	1.00
Raynaud’s phenomenon	0	3 (6)	1 (5)	2 (7)	1.00
Facial erythema	0	12 (26)	9 (47)	3 (11)	0.007
Gottron’s papules	0	5 (11)	5 (26)	0 (0)	0.008
Ulcerations	0	3 (6)	3 (16)	0 (0)	0.06
Trunk rash	0	7 (15)	5 (26)	2 (7)	0.10
No extra-respiratory manifestations	0	17 (36)	6 (31)	11 (39)	0.81

*aMDA*-*5* anti-MDA-5 antibodies, *AS* anti-synthetase, *ARF* acute respiratory failure, *HIV* human immunodeficiency virus, *ICU* intensive care unit, *IQR* inter-quartile range, *SAPS2* simplified acute physiology score, *SOFA* sepsis-related organ failure assessment

### Laboratory and radiological findings

Biological data at the time of ICU admission and radiological findings are reported in Table 2. C-reactive protein levels (*N* < 5 mg/L) were increased (139 [40–208] mg/L), while procalcitonine levels (*N* < 0.5 ng/mL) were not (0.30 [0.12–0.56] ng/mL). The rate of patients having creatine kinase plasma levels greater than 2 times the upper limit of normal laboratory range was 20% (*n* = 9/47) in the whole population, and only 31% (*n* = 8/28) in the AS group. The median PaO_2_/FiO_2_ ratio at ICU admission was 123 [83–147] mmHg.

**Table 2:** Biological, radiological and cytological findings in patients with acute respiratory failure revealing anti-synthetase syndrome or dermato-pulmonary syndrome associated with anti-MDA-5 antibodies

	Missing data	All patients *N* = 47	aMDA-5 ARF *N *= 19	AS ARF *N *= 28	*p*
Biological data at ICU admission, median [IQR]					
Leukocytes count (10^3^/mm^3^)	1	11.5 [8.5–17]	8.4 [6.7–9.8]	16.0 [12.1–21.1]	< 0.001
C-reactive protein (mg/L)	9	139 [40–208]	38 [22–99]	187 [128–262]	0.30
Procalcitonine (ng/mL)	9	0.30 [0.12–0.56]	0.32 [0.11–0.48]	0.30 [0.13–1.42]	< 0.001
Creatininemia (µmol/L)	14	63 [51–78]	59 [51–73]	64 [51–80]	0.04
Creatine kinase (UI/L)	3	157 [69–256]	127 [75–186]	192 [69–932]	0.76
Creatine kinase ≥ 2*N*	3	9 (20)	1 (6)	8 (31)	0.02
PaCO_2_ (mmHg)	1	34 [31–41]	34 [32–42]	33 [31–41]	0.06
PaO_2_/FiO_2_ (mmHg)	3	123 [83–147]	139 [91–174]	117 [78–144]	0.82
Chest X-ray, *n* (%)					
Bilateral opacities	0	45 (96)	19 (100)	26 (93)	0.51
Lower lobe location	0	46 (98)	19 (100)	27 (96)	1.00
Number of quadrants on chest X-ray, *n*					
1	0	1 (3)	0 (0)	1 (4)	0.30
2		27 (68)	10 (59)	17 (74)	
4		12 (30)	7 (41)	5 (22)	
Chest CT scan, *n* (%)					
Performed	0	47 (100)	19 (100)	28 (100)	1.00
Ground-glass attenuation	0	37 (78)	18 (96)	19 (68)	0.03
Alveolar consolidations	0	35 (75)	13 (68)	22 (79)	0.51
Septal thickening	0	12 (26)	8 (42)	4 (14)	0.05
Pleural effusion	0	13 (28)	3 (16)	10 (36)	0.24
Pneumothorax	0	4 (9)	3 (16)	1 (3.6)	0.29
Pneumomediastinum	0	1 (2)	1 (5)	0 (0)	0.40
Mediastinal lymphadenopathy	0	18 (38)	7 (37)	11 (39)	1.00
Signs of lung fibrosis	0	17 (36)	6 (32)	11 (39)	0.82
Traction bronchiectasis	0	17 (36)	6 (32)	11 (39)	0.82
Reticulations	0	12 (26)	4 (21)	8 (29)	0.78
Honeycombing	0	5 (10)	1 (5)	4 (14)	0.64
Broncho-alveolar lavage (BAL), *n* (%) or median [IQR]					
Performed	0	42 (89)	16 (84)	26 (93)	0.38
Delay ICU admission—BAL	0	1 [0–3]	0 [− 1.5–1.0]	2 [1–4]	0.08
Total cell count (10^3^/mL)	8	250 [140–330]	250 [128–390]	250 [160–290]	0.42
Lymphocytes (%)	1	11 [4–30]	22 [8–34]	5 [3–17]	0.06
Neutrophils (%)	2	38 [13–65]	15 [6–36]	51 [20–80]	0.001
Macrophages (%)	2	40 [20–60]	53 [39–73]	29 [15–83]	0.009
Eosinophils (%)	6	0 [0–2]	0 [0–1]	0 [0–2]	0.154
Presence of siderophages	5	2 (4)	1 (6)	1 (4)	1.00
Lung biopsy, *n* (%)	0	4 (9)	1 (5)	3 (11)	–
Diffuse alveolar damage		1	0	1	
Usual interstitial pneumonitis		1	0	1	
Organizing pneumonia		2	1	1	
Skin biopsy, *n* (%)	0	6 (13)	5 (26)	1 (4)	–
Normal		1	0	1	
Lichenoïd dermatitis		3	3	0	
Dermatomyositis		2	2	0	
Muscle biopsy, *n* (%)	0	7 (15)	4 (21)	3 (11)	–
Inflammatory myositis		7	4	3	

*aMDA*-*5* anti-MDA-5 antibodies, *AS* anti-synthetase, *ARF* acute respiratory failure, *BAL* broncho-alveolar lavage, *ICU* intensive care unit, *IQR* inter-quartile range

Most patients (*n* = 45/47, 96%) had bilateral condensations on chest X-ray, with a predominantly lower location (*n* = 46/47, 98%) (Table 2). All patients underwent a lung CT scan, which showed ground-glass attenuation in 78% (*n* = 37/47) and alveolar condensation in 75% (*n* = 35/47). Signs of lung fibrosis were observed in 36% (*n* = 17/47), while 38% (*n* = 18/47) had mediastinal lymphadenopathies.

### Broncho-alveolar fluid analysis and histological data

BAL fluid analyses were available in 89% (*n* = 42/47) of patients and are summarized in Table 2. The cell count was 250 [140–330] × 10^3^/mL, and percentages of lymphocytes, neutrophils and macrophages were 11% [4–30], 38% [13–65] and 40% [20–60], respectively. BAL was performed before antibiotic therapy in only 12/42 (29%) patients and was negative for lung infection in every patient. There was no correlation between BAL findings and elementary lesions observed on chest CT scan. In particular, the proportion of patients with > 40% BAL neutrophils did not differ between patients with or without elementary lesions of lung fibrosis on chest CT scan (*n* = 8/19, 42% vs. *n* = 11/19, 58%, *p* = 0.72). An open lung biopsy was performed in 4 (9%) patients and depicted findings consistent with organizing pneumonia (*n* = 2), usual interstitial pneumonitis (*n* = 1) and diffuse alveolar damage (*n* = 1) (Table 2). A total of 13 patients (28%) had a muscle (*n* = 7) or a skin (*n* = 6) biopsy performed during the ICU stay. All muscle biopsies revealed findings consistent with an inflammatory myositis, while skin biopsies were either normal (*n* = 1) or revealed findings consistent with lichenoid dermatitis (*n* = 3) or with dermatomyositis (*n* = 2) (Table 2).

### ICU management and outcome

Most patients (*n* = 41/47, 89%) received an antimicrobial therapy upon ICU admission (Table 3). All patients received steroids, after a median delay of 6 [3–12] days following the ICU admission. Other immunosuppressive treatments administered are reported in Table 3.

**Table 3:** ICU management and outcome of patient with acute respiratory failure revealing anti-synthetase syndrome or dermato-pulmonary syndrome associated with anti-MDA-5 antibodies

	Missing data	All patients *N *= 47	aMDA-5 ARF *N *= 19	AS ARF *N *= 28	*p*
Treatment, *n* (%) or median [IQR]					
Antibiotics therapy at ICU admission	0	41 (89)	15 (79)	26 (96)	0.14
Immunosuppressive treatment	0	47 (100)	19 (100)	28 (100)	1.00
Delay ICU-IS treatment (days)	0	6 [3–12]	4 [1.5–11]	6 [4–16]	0.25
Corticosteroids pulses	0	47 (100)	19 (100)	28 (100)	1.00
Cyclophosphamide	0	34 (72)	16 (84)	18 (64)	0.24
Rituximab	0	7 (15)	6 (32)	1 (4)	0.01
Cyclosporine	0	2 (4)	2 (10)	0 (0)	0.16
Tacrolimus	0	2 (4)	2 (11)	0 (0)	0.16
Basiliximab	0	2 (4)	2 (11)	0 (0)	0.16
Intravenous immunoglobulins	0	10 (21)	5 (26)	5 (18)	0.50
Plasma exchange	0	8 (17)	7 (37)	1 (4)	0.005
Number of immunosuppressive treatments	0	2 [2, 3]	3 [2–4]	2 [1, 2]	< 0.001
Ventilatory support, *n* (%)					
Noninvasive ventilation before intubation	0	14 (30)	4 (21)	10 (36)	0.45
High-flow nasal cannula oxygen before intubation	0	21 (45)	11 (58)	10 (36)	0.23
Tracheal intubation	0	43 (92)	18 (95)	25 (89)	0.64
ARDS	0	42 (89)	18 (95)	24 (86)	0.64
Mild (200 < PaO_2_/FiO_2_ ≤ 300 mmHg)	0	1 (2)	1 (6)	0 (0)	0.43
Moderate (100 < PaO_2_/FiO_2_ ≤ 200 mmHg)	0	5 (12)	0 (0)	5 (21)	0.06
Severe (PaO_2_/FiO_2_ ≤ 100 mmHg)	0	36 (86)	17 (94)	19 (79)	0.21
Nitric oxide inhalation	0	23 (50)	14 (74)	9 (33)	0.02
Neuromuscular blocking agents	0	36 (78)	17 (90)	19 (70)	0.16
Prone position	0	26 (57)	12 (63)	14 (52)	0.64
Veno-venous ECMO	0	8 (17)	6 (32)	2 (7)	0.05
Extra-ventilatory support, *n* (%)					
Renal replacement therapy	0	8 (17)	4 (21)	4 (14)	0.70
Vasopressors	0	37 (79)	17 (90)	20 (71)	0.17
Outcome, *n* (%) or median [IQR]					
Duration of invasive mechanical ventilation (days)	0	21 [14–35]	19 [9–26]	23 [17–37]	0.34
Length of ICU stay (days)	0	26 [19–38]	22 [17–36]	28 [21–38]	0.20
Length of hospital stay (days)	0	38 [22–67]	22 [19–44]	44 [33–70]	0.015
ICU mortality	0	21 (45)	16 (84)	5 (18)	< 0.001
Hospital mortality	0	24 (51)	16 (84)	8 (29)	0.001

*aMDA*-*5* anti-MDA-5 antibodies, *AS* anti-synthetase, *ARDS* acute respiratory distress syndrome, *ARF* acute respiratory failure, *ECMO* extra-corporal membrane oxygenation, *ICU* intensive care unit, *IQR* inter-quartile range, *IS* immunosuppressive treatment

Almost all patients (*n* = 42/47, 89%) had ARDS, categorized as severe (PaO_2_/FiO_2_ ≤ 100 mmHg with PEEP ≥ 5 mmH_2_O) in 86% (*n* = 36/47), with 17% (*n* = 8/47) of them requiring ECMO. ICU and hospital mortality rates were 45% (*n* = 21/47) and 51% (*n* = 24/47), respectively. Patients with aMDA-5 dermato-pulmonary syndrome had a higher ICU mortality than those with AS syndrome (*n* = 16/19, 84% vs. *n* = 5/28, 18%; *p* < 0.001). Among the 26 ICU survivors, 5 (19%) were diagnosed with a cancer (colorectal *n* = 3, pharyngeal *n* = 1, melanoma *n* = 1) during the 279 [158–500] days post-ICU stay follow-up.

### Comparison between hospital survivors and non-survivors

**Fig. 1:**
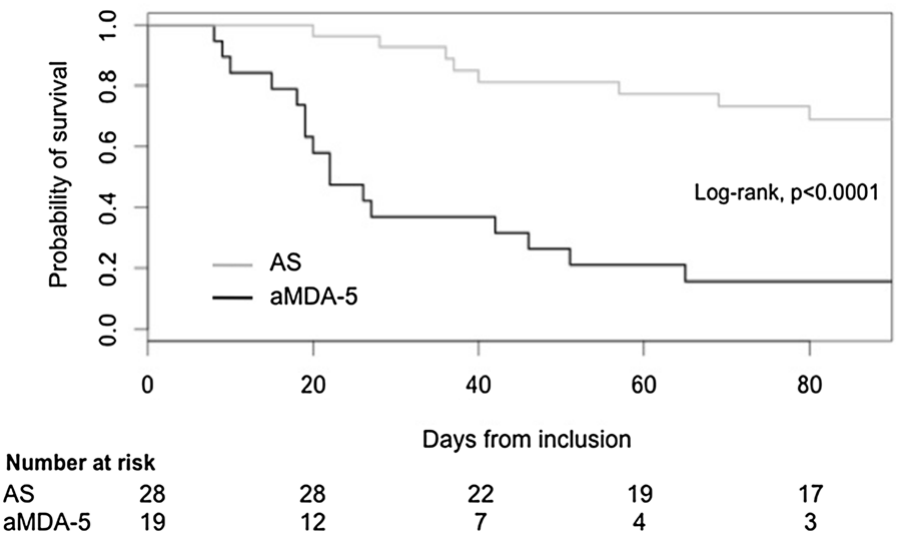
Kaplan–Meier graph of the probability of survival from ICU admission to day 90 in patients with dermato-pulmonary syndrome associated with anti-MDA-5 antibodies (black curve) and patients with anti-synthetase syndrome (gray curve)

Compared to patients who survived at the hospital discharge, those who died were more likely to have an aMDA-5 autoantibody (*n* = 16/24, 67% vs. *n* = 3/23, 13%; *p* = 0.001), had a higher rate of ground-glass attenuation (*n* = 22/24, 92% vs. 15/23, 65%; *p* = 0.04) and a lower rate of alveolar condensation (*n* = 14/24, 58% vs. 21/23, 91%; *p* = 0.02) on chest CT scan, and were given 3 [2, 3] versus 2 [1, 2] different immunosuppressive regimens during the ICU stay (*p* = 0.002) (Table 4). After adjustment on syndrome (anti-synthetase or aMDA-5 dermato-pulmonary syndrome), the presence of ground-glass attenuations on chest CT scan was no longer associated with in-hospital mortality (*p* = 0.24). The Kaplan–Meier graph showed a lower probability of survival 90 days after ICU admission in patients with aMDA-5 antibody than in patients with AS antibody (Fig. 1; *p* < 0.0001 log-rank test).

**Table 4:** Comparison between hospital survivors and non-survivors

	Non-survivors *N *= 24	Survivors *N *= 23	*p*
Clinical, biological and immunological characteristics, *n* (%) or median [IQR]			
Age	65 [59–70]	55 [50–64]	0.062
Male	9 (38)	14 (61)	0.19
SOFA	5 [2–8]	5 [3–8]	0.73
SAPSII	32 [28–53]	41 [27–54]	0.82
Type of autoantibodies			
Anti-MDA-5	16 (67)	3 (13)	0.001
Anti-synthetase antibody	8 (33)	20 (87)	0.001
JO-1	3 (13)	10 (44)	0.04
PL7	3 (13)	6 (26)	0.28
PL12	1 (4)	3 (13)	0.35
EJ	1 (4)	1 (4)	1
Delay first respiratory sign—ICU admission, days	21 [10–43]	20 [10–39]	0.31
Creatine kinase ≥ 2*N*	3 (13)	6 (29)	0.27
PaO_2_/FiO_2_ upon ICU admission	126 [90–149]	117 [82–147]	0.65
Chest X-ray and CT scan, *n* (%)			
Number of quadrants on chest X-ray			
1	1 (5)	0 (0)	0.74
2	13 (62)	14 (74)	
4	7 (33)	5 (26)	
Ground-glass attenuation on chest CT scan	22 (92)	15 (65)	0.04
Alveolar consolidations on chest CT scan	14 (58)	21 (91)	0.02
Signs of lung fibrosis on chest CT scan	9 (38)	8 (35)	1.00
Broncho-alveolar lavage (BAL), *n* (%) or median [IQR]			
Total cell count (10^3^/mL)	250 [80–370]	260 [189–293]	0.36
Lymphocytes percentage	17 [5–31]	7 [3–18]	0.33
Lymphocytes > 10%	12 (55)	9 (45)	0.76
Lymphocytes > 25%	8 (36)	4 (20)	0.41
Neutrophils percentage	20 [10–52]	49 [18–73]	0.12
Neutrophils > 40%	8 (38)	11 (55)	0.44
Neutrophils > 65%	3 (14)	7 (35)	0.16
Management in ICU, *n* (%) or median [IQR]			
Immunosuppressive (IS) treatment			
Delay ICU admission—IS treatment	4 [3–12]	6 [3–16]	0.63
Number of IS treatments	3 [2, 3]	2 [1, 2]	0.002
Corticosteroids	24 (100)	23 (100)	1.00
Cyclophosphamide	20 (83)	14 (61)	0.16
Rituximab	6 (25)	1 (4)	0.10
Basiliximab	2 (8)	0 (0)	0.49
Cyclosporine	1 (4)	1 (4)	1.00
Tacrolimus	2 (8)	0 (0)	0.19
Intravenous immunoglobulins	7 (29)	3 (13)	0.29
Plasma exchange	6 (25)	2 (9)	0.25
Tracheal intubation	24 (100)	19 (83)	0.05
ARDS	23 (96)	19 (83)	0.19
Severe (PaO_2_/FiO_2_ ≤ 100 mmHg)	22 (96)	14 (74)	0.07
Moderate (100 < PaO_2_/FiO_2_ ≤ 200 mmHg)	1 (4)	4 (21)	0.16
Mild (200 < PaO_2_/FiO_2_ ≤ 300 mmHg)	0 (0)	1 (5)	0.45
Nitric oxide inhalation	18 (78)	5 (22)	< 0.001
Veno-venous ECMO	6 (25)	2 (9)	0.25
Vasopressors	21 (88)	16 (70)	0.17
Renal replacement therapy	5 (21)	3 (13)	0.70

*ARDS* acute respiratory distress syndrome, *CI* confidence interval, *ECMO* extra-corporeal membrane oxygenation, *ICU* intensive care unit, *IQR* inter-quartile range, *IS* immunosuppressive, *OR* odds ratio

## Discussion

We are herein reporting the first large cohort of patients admitted to ICU for ARF revealing either AS or aMDA-5 dermato-pulmonary syndrome. The main findings are: (1) clinical manifestations may be nonspecific with the absence of extra-pulmonary manifestations of inflammatory myositis in one-third of patients; (2) hypoxemia is severe with a high rate of severe ARDS and rescue maneuvers; and (3) hospital mortality is high, especially in dermato-pulmonary syndrome associated with aMDA-5 autoantibodies.

AS and aMDA-5-associated dermato-pulmonary syndromes are two near each of the other inflammatory myopathies that may be responsible for severe acute interstitial lung diseases [9–11]. The diagnosis is easy to consider when extra-pulmonary manifestations are present. In AS syndrome, the main extra-pulmonary manifestations include myositis with elevated creatine kinase levels, non-erosive arthritis, Raynaud’s phenomenon and thick cracked skin over the tips and sides of the fingers called “mechanic’s hands” [27–32]. However, there is a wide heterogeneity in clinical manifestations depending on the causative AS autoantibody [33, 34]. In aMDA-5-associated dermato-pulmonary syndrome, the cutaneous manifestations (skin ulcerations or necrosis, facial erythema, mechanic’s hands, periungual telangiectasia, Gottron’s papules, Raynaud’s phenomenon) are in the forefront [10, 11, 35] and usually contrast with the absence of clinical signs of myositis (clinically “amyopathic myositis”). Demographical and clinical findings in our patients were in line with those recently reported in non-ICU patients with AS [22, 32, 34] or with aMDA-5 dermato-pulmonary syndromes [10].

Both in AS and aMDA-5 dermato-pulmonary syndromes, extra-pulmonary manifestations may be lacking [9, 10] rendering the diagnosis difficult to make. In our series, more than one-third of patients had no extra-pulmonary manifestations with a similar proportion in AS and aMDA-5 patients. This rate contrasts with the 10% rate recently reported [10] in patients with aMDA-5 dermato-pulmonary syndrome, reflecting the lack of training of intensivists for the clinical assessment of these patients and highlighting the need for a multidisciplinary approach. Considering the high proportion of patients lacking extra-pulmonary manifestations, the clinical presentation may mimic that of a “bilateral pneumonia without microbiological documentation.” Hence, 89% of our patients received antibiotic therapy at ICU admission. The presence of an intense inflammatory syndrome with increased C-reactive protein levels contrasting with the lack of elevation of serum procalcitonine could help intensivists appreciating the probability of an infectious process, this dissociation being highly suggestive of a non-infectious inflammatory process.

In our series, BAL was performed in 89% of patients. Unlike a recent work [3] showing that a lymphocytic BAL fluid was associated with better ICU survival in ARDS patients with no common risk factor, our study failed to identify any predictive role of BAL cytology on hospital survival. BAL fluid analysis does not seem a useful diagnostic tool for AS or aMDA-5 dermato-pulmonary syndromes, but should nevertheless be performed to rule out an alternative diagnosis, such as diffuse alveolar hemorrhage or active infection.

All included patients underwent chest CT scan. Interestingly, CT chest findings predominate in the lower lobes, which is consistent with a previous report [36]. CT scan signs of lung fibrosis have been recently shown to be associated with a poor outcome in patients with ARF related to interstitial lung diseases [37]. In our study, CT scan signs of lung fibrosis were not associated with hospital mortality, probably because of a lack of adequate power. While ground-glass opacities are usually considered as potentially reversible lung lesions during idiopathic pulmonary fibrosis [38, 39], these lesions were associated with in-hospital mortality in our study, probably because they were more frequently observed during aMDA-5 dermato-pulmonary syndromes. Indeed, this association was no longer observed after adjustment on the type of positive antibody (anti-synthetase or aMDA-5).

Our series underlines the severity of AS and aMDA-5 dermato-pulmonary syndrome, since 89% of patients fulfilled the Berlin criteria for ARDS [5], categorized as severe (PaO_2_/FiO_2_ ≤ 100 mmHg with PEEP ≥ 5 mmH_2_O) in 86% of cases. Anti-MDA-5 dermato-pulmonary syndromes exhibited a significantly higher mortality than AS syndromes, with almost all these patients dying in the ICU of refractory ARDS despite a high rate of ECMO (32%). Moreover, aMDA-5 patients had a much higher mortality than those with severe ARDS included in the lung safe study [7], highlighting the irreversibility of lung lesions despite immunosuppressive treatments. These results are in line with previous series, showing that refractory ARDS is the leading cause of mortality in aMDA-5 patients [10].

Whether our patients had a true ARDS (i.e., presence of diffuse alveolar damage (DAD), the histological hallmark of ARDS) or simply fulfilled the Berlin criteria while having a non-DAD histology is unknown. In fact, the Berlin definition of ARDS is not fully reliable for diagnosing DAD, and several non-DAD histological entities (such as lung fibrosis, organizing pneumonia, diffuse alveolar hemorrhage or lung tumoral infiltration) have been reported in patients fulfilling the clinical and radiological criteria for ARDS [1, 40–42]. Regarding the onset of lung injury, the Berlin definition of ARDS stipulates that “respiratory signs should occur (or worsen) within 7 days after an exposure to a common ARDS risk factor” (e.g., pneumonia, acute pancreatitis, aspiration of gastric content or extra-pulmonary sepsis). In our patients, the absence of a common risk factor for ARDS according to the Berlin definition together with delay between first respiratory sign and ICU admission exceeding 7 days (21 days) advocate more for an ARDS mimicker rather than for a real ARDS. However, a recent histological study revealed that 50% of patients with an acute decompensation of AS syndrome due to JO-1 autoantibody exhibited histological lesions of DAD [43].

In non-ICU patients, the prognosis of inflammatory myopathies depends on the severity of lung involvement [10, 22, 32, 44]. Treatment of interstitial lung disease associated with AS and aMDA-5 dermato-pulmonary syndromes is not standardized and based on case reports. Numerous immunosuppressive therapies are available (e.g., cyclophosphamide, methotrexate, azathioprine, mycophenolate mofetil, cyclosporine, tacrolimus, rituximab, basiliximab, intravenous immunoglobulins or plasma exchange) [9, 11, 14, 21, 45, 46], but high-dose corticosteroids remain the first-line therapy. Our study underlines the wide variations in the choice of immunosuppressive treatment even if the association corticosteroids–cyclophosphamide was administered in almost 3 over 4 patients. Patients with aMDA-5 received significantly more immunosuppressive drugs highlighting a higher severity.

Of note, 19% of ICU survivors developed cancer, in line with previous series of AS patients [47].

### Limitations

Our study suffers from several limitations. First, we included a limited number of patients, inherent to the rarity of the disease. However, this is the first series on ARF revealing AS or aMDA-5 syndromes in an ICU context and our findings are consistent with previous reports. This limited number of patients precluded performing multivariable analyses and thus did not allow for adjusting the observed association between some variables and mortality with potential confounders. Second, the relationship between positive AS or aMDA-5 autoantibody and ARF is not proven. We therefore cannot exclude that some patients had a fortuitously positive autoantibody and that inflammatory myopathy was not the cause of ARF. However, this hypothesis appears unlikely since an alternative diagnosis for ARF had to be excluded, and all patients were treated with immunosuppressive therapies underlining the high degree of clinician’s suspicion. Third, because the patients were recruited over a 13-year period in 35 centers, ICU procedures were inevitably heterogeneous. Fourth, the prevalence of aMDA-5 dermato-pulmonary syndromes may have been underestimated during the study period since detection of aMDA-5 autoantibody was first described in 2005 [48] and was therefore routinely available only from 2010 in most of participating centers. Last, several classical predictors of mortality related to ventilation (tidal volume or driving pressure [49]) were not available as a result of a long-term retrospective design.

### Clinical implications

Considering the high proportion of patients lacking extra-pulmonary manifestations and the nonspecific presentation mimicking that of a bilateral community-acquired pneumonia, we believe that ARF related to autoimmune inflammatory myopathies may be underdiagnosed. Hence, de Prost et al. recently showed that the diagnostic work-up performed in ARDS patients with no common risk factor was not comprehensive, with only 5% of patients having immunological tests [4]. The lack of screening for AS or aMDA-5 autoantibodies is probably one of the reasons why these diseases are underestimated. Therefore, when the etiology of ARF appears unclear, we recommend a more aggressive diagnostic work-up [6], including immunological tests in order to identify patients amenable to specific therapies.

A careful assessment of extra-pulmonary manifestations, such as cutaneous or articular signs, is crucial. While the presence of extra-pulmonary manifestations is highly suggestive, the 3-week delay between first respiratory signs and ICU admission, the absence of an obvious etiology for ARF, the presence of bi-basal consolidations on chest X-ray with an intense inflammatory process, contrasting with a low procalcitonin level together with the lack of microbiological documentation are the main clues to consider the diagnosis of AS or aMDA-5 syndromes in a patient without extra-pulmonary manifestation. To better assess the relevance of these signs, further prospective studies aiming at systematically screen for autoantibodies in ARDS without risk factors are needed. Once the diagnosis is made, the management is difficult and requires a multidisciplinary approach involving intensivists, pulmonologists, internists and rheumatologists in order to decide the best-individualized therapeutic strategy.

## Conclusions

Intensivists should consider inflammatory myopathies, such as anti-synthetase syndrome and dermato-pulmonary syndrome associated with anti-MDA-5 antibodies, as a cause of acute respiratory failure when the etiology appears unclear. Extra-pulmonary manifestations are commonly lacking and an isolated lung involvement may reveal the disease. Hospital mortality is high, especially in aMDA-5 dermato-pulmonary syndrome.

## Abbreviations

ARDS: acute respiratory distress syndrome
ARF: acute respiratory failure
AS: anti-synthetase
aMDA-5: anti-MDA-5 autoantibody
BAL: broncho-alveolar lavage
DAD: diffuse alveolar damage
ECMO: extra-corporeal membrane oxygenation
ICU: intensive care unit
